# Endothelin-3 stimulates cell adhesion and cooperates with β1-integrins during enteric nervous system ontogenesis

**DOI:** 10.1038/srep37877

**Published:** 2016-12-01

**Authors:** Elodie Gazquez, Yuli Watanabe, Florence Broders-Bondon, Perrine Paul-Gilloteaux, Julie Heysch, Viviane Baral, Nadège Bondurand, Sylvie Dufour

**Affiliations:** 1 Institut Curie, CNRS, UMR144, Paris, 75005, France; 2 INSERM, U955, Team 6, Créteil, 94000, France; 3 Université Paris Est, Faculté de Médecine, Créteil, 94000, France; 4 INSERM, U955, Team 11, Créteil, 94000, France; 5 Cell and Tissue Imaging Facility, PICT-IBiSA, Paris, 75005, France

## Abstract

Endothelin-3 (EDN3) and β1-integrins are required for the colonization of the embryonic gut by enteric neural crest cells (ENCCs) to form the enteric nervous system (ENS). β1-integrin-null ENCCs exhibit migratory defects in a region of the gut enriched in EDN3 and in specific extracellular matrix (ECM) proteins. We investigated the putative role of EDN3 on ENCC adhesion properties and its functional interaction with β1-integrins during ENS development. We show that EDN3 stimulates ENCC adhesion to various ECM components *in vitro*. It induces rapid changes in ENCC shape and protrusion dynamics favouring sustained growth and stabilization of lamellipodia, a process coincident with the increase in the number of focal adhesions and activated β1-integrins. *In vivo* studies and *ex-vivo* live imaging revealed that double mutants for *Itgb1* and *Edn3* displayed a more severe enteric phenotype than either of the single mutants demonstrated by alteration of the ENS network due to severe migratory defects of mutant ENCCs taking place early during the ENS development. Altogether, our results highlight the interplay between the EDN3 and β1-integrin signalling pathways during ENS ontogenesis and the role of EDN3 in ENCC adhesion.

Development of the enteric nervous system (ENS) starts by the colonisation of the intestine by enteric neural crest cells (ENCCs) that migrate rostrocaudally through the gut mesenchyme and the mesentery. This requires the coordinated regulation of ENCC migration, proliferation, differentiation into neurons and glial cells (for review^1^), and their aggregation to form the ENS ganglia network^2^,^3^,^4^. This process is governed by ENCC-autonomous and non-autonomous mechanisms, including cell responses to environmental cues, including extracellular matrix (ECM) and soluble factors^5^,^6^,^7^, such as endothelin-3 (EDN3)^8^,^9^.

EDN3 is expressed in the developing gut and at high levels in the caecum when ENCCs migrate into this zone^10^. The EDN3/EDN receptor type B (EDNRB) signalling pathway plays a crucial role during ENS development. EDN3 plays an important role in enteric progenitor maintenance and self-renewal^9^,^11^,^12^. It is also involved in ENCC migration^13^,^14^ and contributes to the maintenance of a permissive environment for ENCC colonization^15^,^16^,^17^. *Ednrb*^*−/−*^ and *Edn3*^*ls/ls*^ mutant mice exhibit distal aganglionosis^18^,^19^,^20^ due to defective colonization of the hindgut by ENCCs, which fail to migrate forward through the ileo-caecal junction at E12.5^20^,^21^.

ENCCs express various integrins^22^,^23^,^24^, cell surface receptors that control adhesion to the ECM and many cellular behaviours, including migration, proliferation, survival, and the control of stem cell fate^25^,^26^,^27^. They are heterodimeric receptors composed of one α and one β chain. There are 18 α and 8 β subunits that associate to form 24 different integrin heterodimers^26^. This association determined the recognition of specific ECM components. The β1-integrins represent the largest subfamily, as the β1 chain can associate with 12 different α subunits (α1 to α11 and αV). β1-integrins regulate ENCC colonization of the gut and act in cooperation with N-cadherin to control proper ENS network organisation^24^,^28^. The conditional invalidation of *Itgb1* gene encoding the β1-subunit of integrins in NCCs produces aganglionosis of the distal colon underscoring the requirement for β1-integrin functions during ENS ontogenesis^24^. β1-integrin-null ENCCs display defective migration at the entry of the caecum, a region that is enriched in fibronectin (FN) and tenascin-C (TNC) at the timing of its colonization by ENCCs^5^. The phenotype of conditional *Itgb1* mutants^24^ resembles that described for *Ednrb*^*−/−*^ and *Edn3*^*ls/ls*^ mice. The perturbation of EDNRB signalling through the action of the selective ENDRB antagonist, BQ788, in *ex vivo*-embryonic gut cultures produces a rounding up of ENCCs and modifications of their migratory behaviour^29^,^30^. These results suggest that the possible interplay between integrins, ECM, and EDN3 signalling controls ENCC adhesion and migration during ENS development.

Here, we have investigated the effects of EDN3 on ENCC adhesion properties and the possible interplay between β1-integrins and EDN3/EDNRB signalling during ENS development.

## Results

### EDN3 increases ENCC adhesion and the number and size of focal adhesions

**Figure 1:**
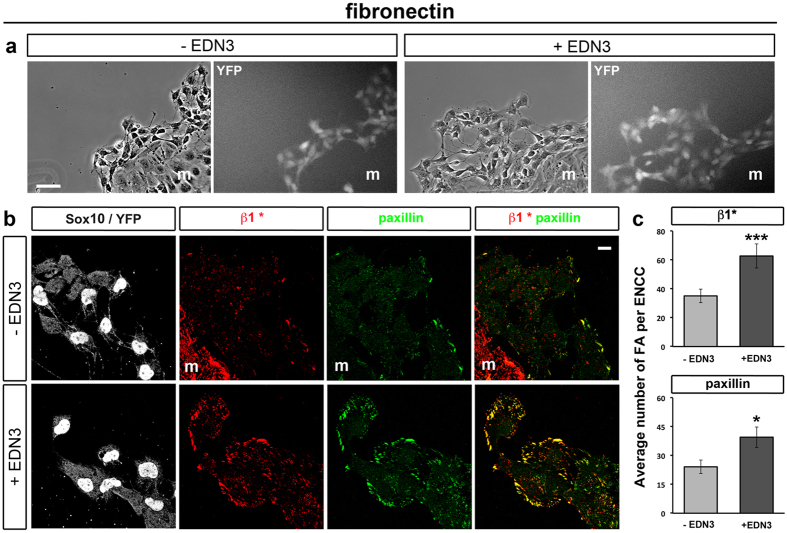
Effect of EDN3 on ENCC adhesion. (**a**) Phase contrast (left panels) and YFP images (right panels) of control E12.5 gut explants cultured 24 h on FN with or without EDN3. (**b**) Confocal images of cultures immunolabeled for Sox10 and YFP (ENCCs, left panels), and for activated β1-integrin (β1*) and paxillin to visualize FAs. When present the letter m refers to mesenchymal cells (Sox10^−^/YFP^−^), which exit the explant together with ENCCs. Merged images for β1* and paxillin are shown in the right panels. (**c**) Average number of β1*- and paxillin-positive FAs per ENCC. The number of cells analysed for β1* staining was n = 508 and 449 and for paxillin staining n = 449 and 310, under control and EDN3 conditions, respectively. Error bars indicate the SEM; *p < 0.05; ***p < 0.001. Scale bar in a and b = 50 and 10 μm, respectively.

We analysed the impact of EDN3 on ENCC adhesion by culturing E12.5 mouse midgut explants for 24 hours on FN, a permissive ECM protein for cell adhesion and migration, with or without EDN3. These gut explants were collected from Ht-PA::Cre;*beta1*^*fl/fl*^;R26RYFP embryos^5^ (see Materials and Methods and Table 1). In these embryos, Ht-PA promoter drives the expression of YFP reporter protein^31^ and the Cre-dependent recombination *Itgb1*-floxed allele specifically in migratory NCC, which are heterozygous for *Itgb1* and considered as control^5^,^32^. ENCCs usually escaped first from the gut explant followed by mesenchymal cells, and were easily distinguished by their morphology and YFP expression. Although they migrated properly at the periphery, the global morphology of ENCC clusters appeared to be more spread-out in the presence of EDN3 (Fig. 1a) and more ENCCs displayed a large lamellipodium than untreated cells. EDN3 treatment clearly increased both activated β1-integrin (β1*integrin; ECM receptor) and paxillin (FA marker) signals at the ENCC periphery (Fig. 1b). Quantification of focal adhesions (FAs) developed by ENCCs showed that EDN3 significantly increased their number relative to the control counterparts (Fig. 1c). The mean FA area and Feret’s diameter (indicative of their length) were significantly increased by EDN3 treatment (Table S1).

Next, we performed the same analysis on cultures performed on FN + TNC (Fig. S1), a protein which is strongly expressed in the caecum and perturbs ENCC adhesion^5^. EDN3 strongly increased the number of ENCC FAs on FN + TNC. Quantification of their average number per ENCC revealed a two-fold increase of FAs in EDN3-treated ENCCs relative to untreated cells. ENCCs cultured on FN + TNC exhibited smaller FAs than cells cultured on FN alone. EDN3 slightly and significantly increased FA size, as observed for cells cultured on FN alone (Table S1).

Finally, we tested whether EDN3 also increases ENCC adhesion to vitronectin (VN), another permissive protein expressed in the embryonic gut mesenchyme. ENCCs displayed fewer and smaller FAs on VN than on FN. EDN3 significantly enhanced the average number of FAs per ENCC (Fig. S2a,b) and increased their size (Table S1). VN is recognized by several integrin receptors including αVβ1 and αVβ3 integrins. Unlike for β1*integrin, EDN3 did not modify β3-integrin-positive adhesion sites on this substrate. Indeed, very few β3-integrin signals were detected in either control or EDN3-treated cultures (Fig. S2c).

Altogether, our results indicate that EDN3 enhances the adhesive capacity of ENCCs to various ECM proteins expressed in the embryonic gut through the stimulation of the number and size of FAs.

### EDN3 induces a rapid change in ENCC morphology

**Figure 2:**
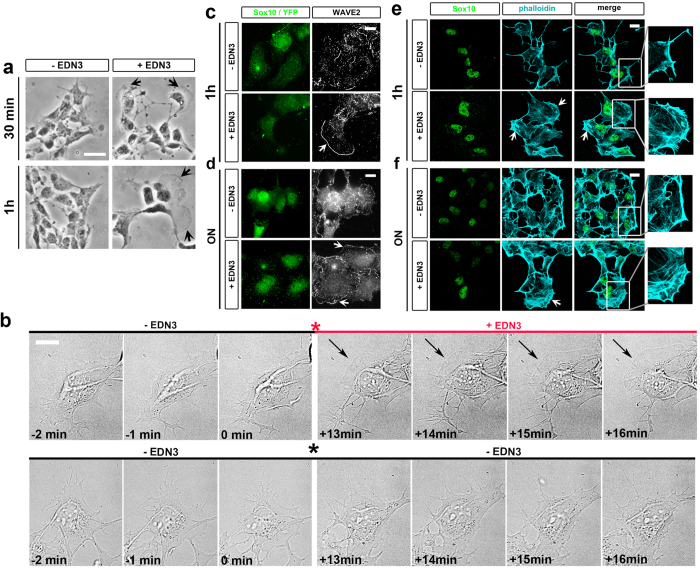
EDN3 favours a sustained growth of ENCC protrusions. (**a**) Phase contrast images showing spread ENCCs with lamellipodia (black arrows) on FN after 30 min or 1 h of EDN3 treatment, or in control medium. Scale bar = 50 μm. (**b**) Images extracted from a time-lapse movie of gut explant cultures in control medium immediately before, and 13 min after, the change of medium (indicated by an asterisk) for medium with (red line) or without (black line) EDN3. The upper and lower right panels in b were extracted from the movies 1 and 2 (see supplementary Videos), respectively. After stimulation with EDN3, a lamellipodium start to grow on the ENCC (black arrows, upper panels), but not after incubation with control medium (lower panels) for the same period. (**c**,**d**) lamellipodium edge of ENCCs (Sox10^+^/YFP^+^) stained with WAVE2 antibody (white arrows) after a 1 h (**c**) or overnight (ON, **d**) EDN3 treatment. (**e**,**f**) Confocal images showing ENCCs (Sox10^+^/YFP^+^), F-actin (phalloidin) staining, and showing the reorganisation of the actin cytoskeleton at the lamellipodium edge (white arrows) after an overnight (**e**) or 1 h EDN3 treatment (**f**). The organisation of the actin cytoskeleton is shown at a higher magnification in the inserts. Scale bars in (**b–e**) = 10 μm.

We analysed the kinetics of the response of ENCCs to EDN3 to further investigate whether the effect is direct or indirect. We determined the time course of the EDN3′ response in mouse (Fig. 2) and chick midgut cultures (Fig. S3). The explants were cultured on FN in control medium overnight before stimulation by EDN3 for various times. The change in the morphology of ENCCs we previously observed after 24 h of EDN3 treatment was also visible when cultures were treated for only 30 min and one h, whereas it was not observed in their control counterparts (Fig. 2a, arrows, and Fig. S3). We analysed the morphological changes of ENCCs after short EDN3 treatment, especially protrusion dynamics, using time-lapse imaging of both mouse (movies 1 to 2; Fig. S4) and chick cultures. After overnight culture on FN, and before adding the EDN3, ENCCs exhibited rapid membrane dynamics, producing numerous filopodia, ruffles, and unstable lamellipodia (Fig. 2b). We observed that 10–15 minutes after the medium was replaced, 21% of mouse ENCCs and 72% of chick ENCCs displayed a large lamellipodium, which was stable for several minutes (number of cells analysed: 261 and 38 for mouse and chick, respectively). In contrast, there was no change in membrane dynamics when the medium was replaced with control medium containing the solvent (Fig. 2b) (number of cells analysed: 193 and 49 for mouse and chick, respectively). This indicates than EDN3 rapidly modulates cell morphology changes and membrane protrusion dynamics of ENCCs.

### EDN3 induces sustained growth of ENCC lamellipodia through actin cytoskeleton remodelling

Lamellipodia formation is under the control of branched actin polymerization at the cell leading edge, a process requiring the ARP2/3 complex, nucleating factors such as WAVE^33^, and the RHO family of small GTPases^34^. We therefore compared the distribution of WAVE2 and the actin cytoskeleton between untreated ENCCs and those treated with EDN3 for one h and overnight on FN. We detected WAVE2 staining at the periphery of lamellipodia under both conditions. However, we observed more ENCCs exhibiting largely spread lamellipodia with bright WAVE2 staining in cultures treated overnight or for one hour with EDN3 (Fig. 2c,d, white arrows) than under control conditions (Fig. 2c,d). In addition, control ENCCs exhibited cortical actin (Fig. 2e,f), whereas the actin cytoskeleton of EDN3-treated ENCCs was remodelled in both one hour and overnight cultures, with less cortical actin staining and a larger branched actin network at the lamellipodia edges (Fig. 2e,f, white arrows). Similar results on F-actin organisation were observed in chick ENCCs after treatment (Fig. S3).

Altogether, our results show, for the first time, that EDN3 elicits the rapid and sustained growth of ENCC lamellipodia, probably through an ARP2/3-WAVE2-dependent mechanism that triggers actin cytoskeleton remodelling at the cell edge.

### Kinetics of EDN3 effect on ENCC FAs

**Figure 3:**
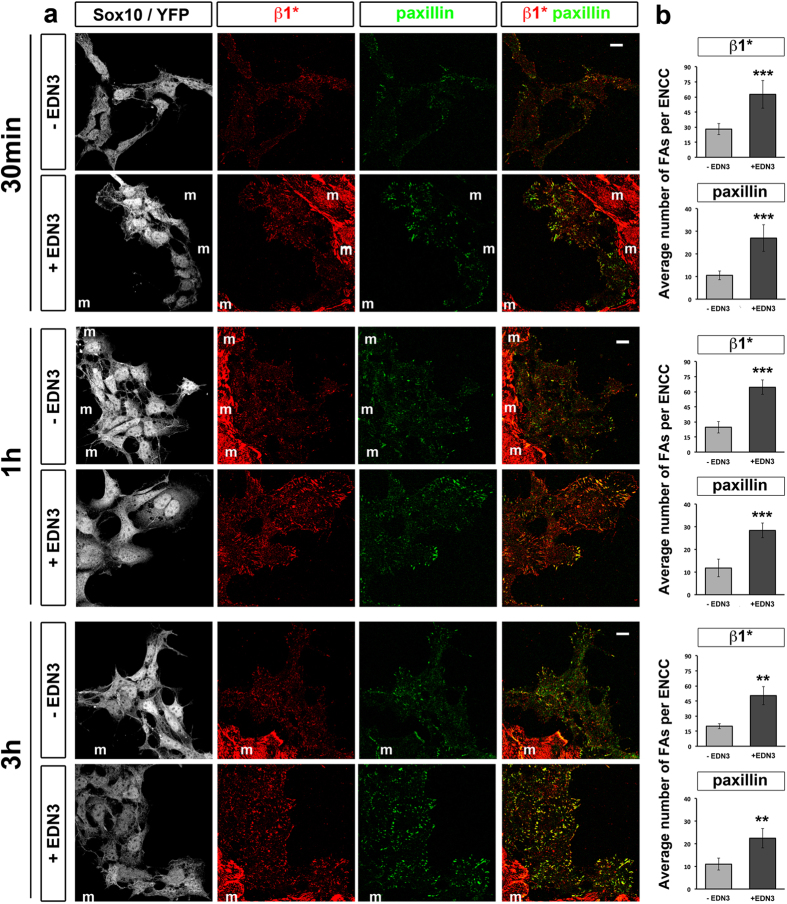
Time-course of the response of ENCCs to short exposure to EDN3. (**a**) Confocal images of control gut explants cultured overnight on FN in control medium prior to EDN3 treatment for 30 min, 1 h or 3 h, immunolabeled for Sox10 and YFP (ENCCs), activated β1-integrin (β1*), and paxillin. The letter m refers to mesenchymal cells (Sox10^−^/YFP^−^). Merged images for β1* and paxillin are shown in the right panels. (**b**) Average number of β1*- and paxillin-positive FAs per ENCC after treatment with or without EDN3 for 30 min, 1 h, and 3 h. The number of cells analysed under control and EDN3 conditions was n = 277 and n = 208 after 30 min; n = 382 and n = 255 after 1 h; and n = 355 and n = 327 after 3 h of treatment, respectively. Error bars indicate the SEM; **p < 0.01; ***p < 0.001. Scale bars = 10 μm.

The growth of lamellipodia is linked to the connection between integrin-dependent adhesion and the actin cytoskeleton^35^. We thus analysed the FAs formed by ENCCs on FN after stimulation by EDN3 for 30 min, one, and three hours. Shorter EDN3 treatment also increased the number of both β1*integrin- and paxillin-positive FAs in mouse (Fig. 3) and chick ENCCs (Fig. S3). Indeed, we observed a more than a two-fold increase in the average number of FAs per ENCC (Fig. 3) and in the FA size for 30 min and 3 h periods relative to controls (Table S1). After one hour of EDN3 treatment, the Feret’s diameter of the FAs increased slightly relative to controls, but there was no effect on their mean area.

Our results show that EDN3 can increase ENCC adhesion after 30 min of treatment, as revealed by the rapid increase of the β1*integrin signals and the number of FAs displayed by ENCCs. Moreover, this effect is persistent over time.

### Adhesion properties of *Edn3* ^*ls/ls*^ENCCs

**Figure 4:**
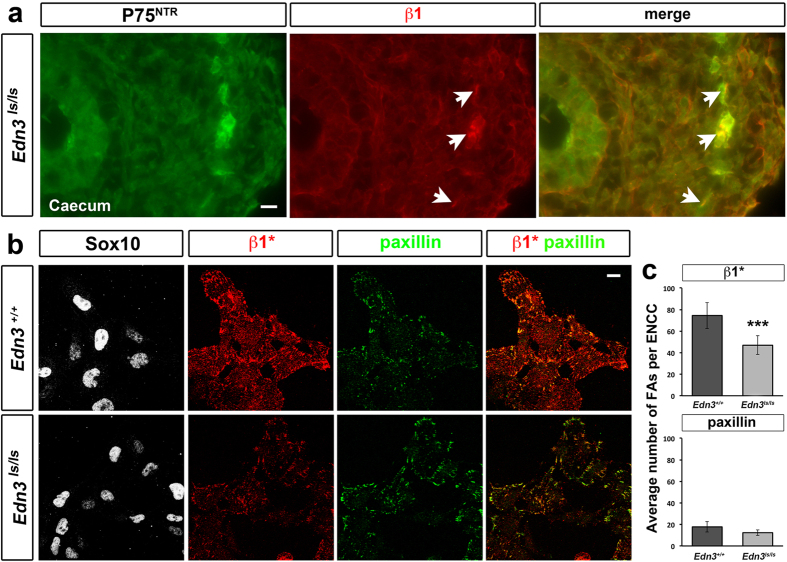
β1-integrin expression and quantification of FAs of *Edn3*^*ls/ls*^ mutant ENCCs. (**a**) Immunostaining of caecum cryosections from E11.5 *Edn3*^*ls/ls*^ mutants (n = 2) for ENCC marker (P75^NTR^) and pan-β1-integrin. Arrows point on β1-integrin staining of ENCCs. A Merged image for P75^NTR^ (green) and β1-integrin (red) is shown in the right panel. (**b**) Activated β1-integrin (β1*) and paxillin immunostaining of ENCC progenitors (Sox10^+^) from E12.5 *Edn3*^+/+^ and *Edn3*^*ls/ls*^ midgut explants cultured 24 h on FN. Scale bars = 10 μm. (**c**) Average number of β1*- and paxillin-positive FAs per ENCC for control and *Edn3*^*ls/ls*^ mutants. The number of cells analysed was n = 836 and n = 711 over 5 *Edn3*^+/+^ and 5 *Edn3*^*ls/ls*^ mutant midguts, respectively. Error bars indicate the SEM; ***p < 0.001.

We analysed the adhesion properties of ENCCs in the context of the *Edn3*^*ls*^ mutation. We first performed cryosections of *Edn3*^*ls/ls*^ midgut that we immunostained for β1-integrin and p75^NTR^ (Fig. 4a). ENCCs (p75^NTR+^) and all other cells of *Edn3*^*ls/ls*^ mutant gut expressed β1-integrins, as previously observed in controls^24^. We then compared the capacity of ENCCs to form FAs on FN. We observed a decrease in signal intensity for β1*integrin in *Edn3*^*ls/ls*^ ENCCs relative to *Edn3*^+*/*+^ cells on FN for 24 h. (Fig. 4b). Quantification of β1*integrin-positive adhesion sites revealed a 37% decrease in the number of FAs displayed by *Edn3*^*ls/ls*^ ENCCs relative to *Edn3*^+*/*+^ cells (Fig. 4c). No significant changes were observed for the FA area (0.197 ± 0.001 and 0.200 ± 0.003 μm^2^ for *Edn3*^*ls/ls*^ and *Edn3*^+*/*+^, respectively) but their Feret’s diameter increased slightly (0.783 ± 0.003 and 0.754 ± 0.002 for *Edn3*^*ls/ls*^ and *Edn3*^+*/*+^, respectively, p < 0.001). There was no difference between the paxillin-positive FAs in the mutant and control ENCCs (Fig. 4b,c).

Our results show that *Edn3*^*ls/ls*^ ENCCs express β1-integrins but recruit less β1-integrins to the FAs relative to control cells.

### *Itgb1* heterozygosity leads to a reduced extent of gut colonization by ENCCs in the context of the *Edn3* ^*ls/ls*^ mutation

It was previously shown that both the *Edn3*^*ls/ls*^ mutant mice, as well as the conditional mutant with NCC-targeted *Itgb1* deletion (Ht-PA-Cre;*beta1*^*neo*/fl^; hereafter referred as β1^null^), display distal aganglionosis^19^,^24^. We investigated whether the depletion of β1-integrin affects EDNRB expression, in addition to perturbing ENCC adhesion and migration properties^5^. Immunostaining of E13.5 control and β1^null^ midgut sections for EDNRB expression revealed that mutant ENCCs expressed similar levels of EDNRB at their surface as control cells (Fig. S5), indicating that they can bind EDN3 present in their environment. *In situ* hybridization experiments showed the presence of *Edn3* mRNA in the β1^null^ embryonic gut as well (unpublished observations).

We then tested the consequences of combined depletion of β1-integrin and EDN3, *in vivo*. We first analysed the impact of introducing the heterozygous *Itgb1* mutation in the context of *Edn3* mutants, because invalidation of the *Itgb1* gene is lethal at the peri-implantation stage^36^. We prepared a series of intercrosses (see Material and Methods) to obtain mutant embryos of six genotypes of interest. For the sake of simplicity, the genotype abbreviations used to describe the mutants of interest are presented in Table 1. We compared enteric phenotype at stage where the complete colonization of the gut by ENCCs would normally occur (E14.5), using whole-mount immunostaining with the neuronal class III β-tubulin TUJ1 marker. Consistent with previous findings^24^,^37^, the heterozygous β1^neo^ and Edn3^het^ guts were fully colonized (Fig. S6). The β1^neo^;Edn3^het^ guts were also fully colonized whereas Edn3^null^ guts showed a delay of colonization revealed by the absence of TUJ1^+^ cells from the distal portion of the small intestine or the caecum. All β1^neo^;Edn3^null^ guts exhibited a colonization defect and four of the seven β1^neo^;Edn3^null^ guts analysed had a more severe phenotype characterized by the absence of TUJ1^+^ cells halfway through the small intestine relative to Edn3^null^ guts.

This suggests that reduction of integrin levels in the context of the *Edn3* mutation influences ENS development.

### ENCC *Itgb1* conditional invalidation in the context of the *Edn3* ^*ls/ls*^ mutation leads to a reduced extent of gut colonization and enteric ganglia network disorganisation

**Figure 5:**
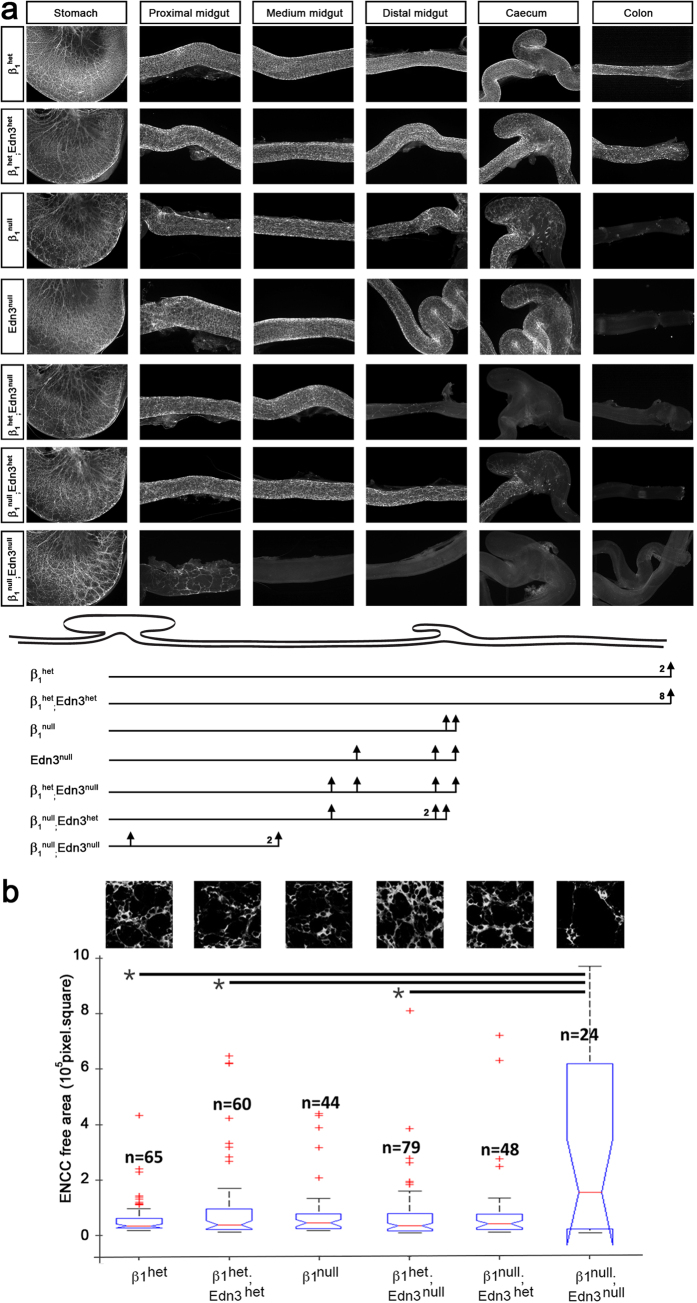
The genetic interaction between *Edn3* and *Itgb1* controls ENS development. (**a**) Images of whole-mount TUJ1 immunostaining of E14.5 guts from β1^het^, β1^het^;Edn3^het^, β1^null^, Edn3^null^, β1^het^;Edn3^null^ β1^null^;Edn3^het^ and β1^null^;Edn3^null^ embryos. A schematic representation of the gut with the lines and perpendicular arrows indicating the extent of colonization for each class of genotyped embryos is shown at the bottom. The number of embryos analysed are indicated to the left of each arrow. (**b**) The ENS network organisation of each class of genotyped embryos. Top panels show confocal compilations of TUJ1 staining taken at the proximal portion of the small intestine. The graph shows the quantification of TUJ1-free areas relative to the mean area summarized as box plots. The top and bottom of each box indicates the 25th and 75th percentiles, respectively. The red line in the middle of the box indicates the median. n indicate the number of TUJ1-free areas analysed.

We then analysed the impact of the targeted conditional deletion of the *Itgb1* gene in NCCs in the context of the *Edn3*^*ls/ls*^ using a double mutant crossing strategy with Ht-PA::Cre mice (see Material and Methods) (Table 1). The enteric phenotypes of these six classes of mutants obtained were compared as described above. We thus observed that the β1^het^ guts were fully colonized whereas the β1^null^ guts exhibited delayed colonization (absence of TUJ1^+^ cells from the caecum, Fig. 5a), consistent with previous findings^24^,^28^,^38^. All the β1^het^;Edn3^het^ guts were fully colonized and were similar to β1^neo^;Edn3^het^ (Fig. S6), but both the β1^het^;Edn3^null^ and β1^null^;Edn3^het^ guts showed a delay of colonization from the distal portion of the small intestine or from the caecum. One of the four guts analysed of β1^het^;Edn3^null^ and β1^null^;Edn3^het^ embryos exhibited a more severe delay of colonization with ENCCs blocked before two-thirds of the small intestine relative to Edn3^null^ and β1^null^ embryos (Figs S6 and 5a). In contrast, all the β1^null^;Edn3^null^ showed more severe delay in colonization relative to single mutants, with TUJ1 staining stopping at the beginning or within the first half of the small intestine in all cases (Fig. 5a).

We also observed disorganisation of the neuronal network with abnormal aggregates surrounded by enlarged TUJ1-free spaces along the β1^null^ guts, as previously described^24^,^28^,^38^. This network disorganisation was greater in β1^null^;Edn3^null^ guts that exhibited larger TUJ1-free regions, but not in other genotypes (Fig. 5a). We quantified the ENCC distribution in the colonized part of the gut in the different genotypes (i.e. the beginning of the small intestine) and measured the ENCC-free areas above the mean size of those of the controls. We observed significantly enlarged ENCC-free areas in β1^null^;Edn3^null^ guts relative to the β1^het^;Edn3^null^, β1^het^;Edn3^het^ and β1^het^ guts (Fig. 5b).

In addition, we compared the organisation of the ENS in the second half of the small intestine in the non-conditional and conditional mutants, with the exception of the β1^null^;Edn3^null^ mutants in which it did not reach this part of the gut. We quantified the texture and performed a principal component analysis (PCA) of the neuronal network (TUJ1^+^) to detect distinct network organisations. The dots for the non-conditional mutant β1^neo^;Edn3^null^ were separated from the other dots by a decision line demonstrating a distinct organisation of the ENS in this mutant relative to the others (Fig. S7). This result demonstrates that *Itgb1* heterozygosity, in the context of the *Edn3* mutation, leads to a significant change in the ENS network that is not observed for EDN3 depletion or β1-integrin depletion alone in ENCCs.

Thus, combined deficits in EDN3 function and β1-integrin-mediated adhesion caused profound ENS defects.

### Timing and cellular origin of ENS defects in double mutants

To determine the origin of the defects we analysed the behaviour of ENCCs earlier during development. Whole-mount X-Gal staining performed on E11.5 guts (Fig. S8) revealed a normal colonization in β1^het^;Edn3^het^ guts, with the ENCC migratory front located at the entry of the caecum, and a slight colonization delay in the β1null 24, and in β1^het^;Edn3^null^ and β1^null^;Edn3^het^ where ENCC front reached the second third of the midgut. In contrast, the β1^null^;Edn3^null^ guts exhibited a severe colonization defect, as revealed by ENCC stopped in the proximal stomach. These results suggest that genetic interaction between *Itgb1* and *Edn3* loci is taking place early during the ENS development.

**Figure 6:**
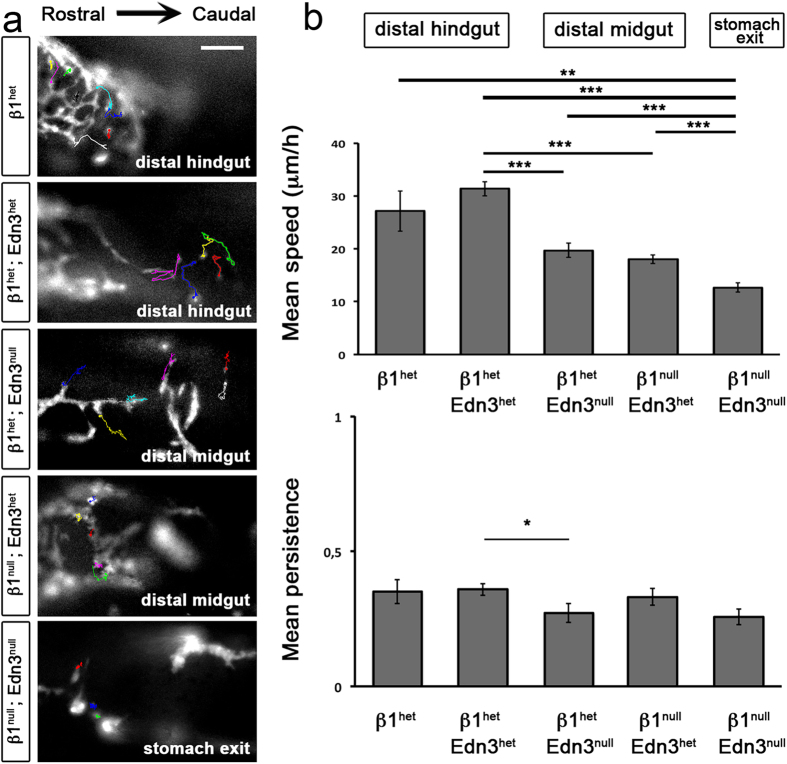
Migratory behaviour of ENCCs within the embryonic gut. (**a**) Images extracted from a representative time-lapse movie of E12.5–13.5 guts cultured *ex-vivo* from β1^het^ (n = 3 guts); β1^het^; Edn3^het^ (n = 7); β1^het^; Edn3^null^ (n = 1); β1^null^; Edn3^het^ (n = 5), and β1^null^; Edn3^null^ (n = 3) genotypes, with examples of individual ENCC trajectories. Scale bar = 100 μm. (**b**) Graphs show the mean ENCC speed of migration depending on the gut genotype (upper graphs) and the mean persistence (lower graphs). The number of ENCCs analysed was: β1^het^ n = 20; β1^het^;Edn3^het^ n = 88; β1^het^;Edn3^null^ n = 21; β1^null^;Edn3^het^ n = 68 and β1^null^;Edn3^null^ n = 37. Error bars indicate the SEM; *p < 0.05; **p < 0.01; ***p < 0.001.

We next investigated the migratory properties of ENCCs in the β1^null^;Edn3^null^ midguts relative to the other genotypes mutants, taking advantage of YFP expression targeted in these cells. We performed time-lapse imaging of *ex-vivo* gut cultures and analysed individual trajectory of YFP^+^ ENCCs at the migratory front to determine their dynamic behaviours within the embryonic gut between E12.5–E13.5. At the beginning of live imaging, ENCC migration fronts were located within different parts of the gut, depending on the genotype analysed. Migratory fronts of both control β1^het^ and β1^het^;Edn3^het^ guts were at the distal hindgut, whereas the migratory fronts of β1^null^;Edn3^het^ and β1^het^;Edn3^null^ were at the distal midgut. No clear migratory front was distinguishable for β1^null^;Edn3^null^ guts; although β1^null^;Edn3^null^ ENCCs reached the stomach exit, more radial portions of the stomach remained uncolonized. Figure 6a presents examples of ENCC trajectories for each genotype. The morphology of β1^null^;Edn3^null^ ENCCs resemble that observed in β1^null^;Edn3^het^ mutants with some isolated ENCCs exhibiting a rounder shape and others exhibiting increased aggregation capacity relative to the other genotypes. The trajectories of β1^null^;Edn3^null^ ENCCs were clearly shorter than for all the other genotypes. The double-mutant ENCCs were motile but with no real migratory capacity and some were static, reflecting their inability to efficiently migrate rostrocaudally or in radial directions. The migration speed of the β1^null^;Edn3^null^ ENCCs was significantly lower than that measured for β1^null^;Edn3^het^ and β1^het^;Edn3^null^ ENCCs and both control β1^het^;Edn3^het^ and β1^het^ ENCCs. The migration speed of β1^null^;Edn3^het^ and β1^het^;Edn3^null^ ENCCs were not significantly different from each other, whereas they were significantly less than that of β1^het^;Edn3^het^ control ENCCs. Finally, β1^het^;Edn3^null^ ENCCs displayed a slight, albeit significant, reduction in the persistence of their migration relative to β1^het^;Edn3^het^ ENCCs, whereas no significant differences were found when the other genotypes were compared to each other (Fig. 6b).

Altogether, our results show a severe migratory defect of double-mutant ENCCs that fail to migrate efficiently through the midgut, thus highlighting the cooperation between *Edn3* and *Itgb1* to control ENCC migration.

## Discussion

**Figure 7:**
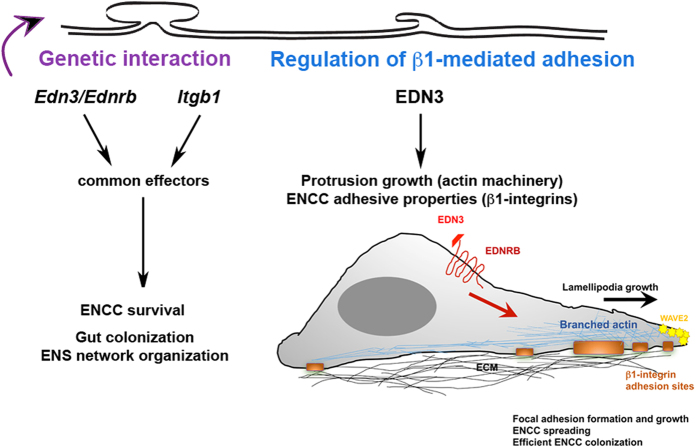
Interplay between the EDN3 and β1-integrin signalling pathways during ENS ontogenesis. Genetic interaction between *Itgb1* and *Edn3* regulates enteric progenitor survival, gut colonization and ENS network organisation. EDN3-dependent signalling promotes sustained growth of lamellipodia, and the formation of new ENCCs-ECM β1-integrin-enriched adhesion sites, two mechanisms required for proper ENS development.

EDN3 is involved in the maintenance of enteric progenitors in an undifferentiated state^11^ and regulate their migration^13^. A relationship between EDN3 and adhesion to the ECM has been demonstrated in astrocytes by stimulating tyrosine phosphorylation of FAK and paxillin^39^, and in cancer cells^40^,^41^. In melanoma cells, EDN3 stimulates the expression of αVβ3 and α2β1 integrins^41^. In the present study, our results highlight the interplay between the EDN3 and β1-integrin signalling pathways during ENS ontogenesis as well as the major role of EDN3, alone and in combination with β1-integrins, in controlling ENCC adhesion (Fig. 7).

Here, we demonstrate a novel role for EDN3 in the adhesion of ENCCs through the increase of FA number and size. We found that the stimulatory effect of EDN3 on cell adhesion is not species-specific as we observed it in both murine and avian ENCCs. Indeed, we observed an EDN3-dependent increase in both activated β1-integrin and paxillin signals at the cell-ECM interfaces. We observed similar increases in chick ENCCs immunostained for pan-β1-integrin or its active state, suggesting that EDN3 does not act directly on integrin activation but rather influences signalling cascade favouring FA formation or maturation. The EDN3 effect on ENCC adhesion occurs rapidly, as it is observed within less than 30 min of stimulation, and is retained for longer periods of treatment. This supports a direct effect on ENCC adhesion rather than an indirect effect related to the activity of EDN3 in maintaining a progenitor state through the regulation of gene expression. EDN3 increased the formation of β1*integrin- and paxillin-positive FAs, but we also observed some β1-integrin-positive FAs with faint or undetectable paxillin staining. Future studies should focus on analysing the impact of EDN3 on the recruitment of other proteins of the integrin adhesome^42^.

In line with the observed increase in FAs shortly after EDN3 treatment, EDN3 rapidly stimulated sustained growth of lamellipodia and changes in actin cytoskeleton remodelling which is known to favour the formation and growth of FAs. This suggests a role of EDN3 in cell polarization, which is important for directional cell migration. Our observation is in agreement with previous findings^13^ showing that treatment of gut explants with EDN3 rapidly increases RAC activation, a small GTPase of the RHO family which is known to regulate actin cytoskeleton remodelling and stimulate the branched actin network responsible for lamellipodia formation^43^,^44^. We observed an increase in lamellipodia and WAVE2-positive protrusions in ENCCs stimulated with EDN3. In agreement with this observation, glioblastoma stem cells treated with the EDNRB antagonist BQ788 displayed downregulation of the *WASF2* gene that encodes WAVE2^45^. These results collectively suggest a role for WAVE2 as a downstream effector of EDN3-mediated regulation of ENCC adhesion, cytoskeleton remodelling, and membrane dynamics.

EDN3-dependent signalling was shown to counteract the inhibitory effect of TNC on glioblastoma cell adhesion to FN^40^. On VN, TNC has an inhibitory effect on ENCC adhesion and migration while FN stimulates these processes^5^. Here, ENCCs exhibited a similar number of FA when cultured on FN alone or on FN + TNC, but they were smaller in size in the latter case. We show that EDN3 can equally stimulate ENCC adhesion properties in FN and FN + TNC-coated surfaces. The effect of EDN3 on glioblastoma cells is dependent on α5β1integrin and syndecan4^40^. ENCCs express α5β1integrin and syndecan4 but treatment with heparin does not impede their adhesion to FN (unpublished observations) suggesting that syndecan4 does not play a crucial role in ENCC adhesion to FN and that the EDN3-dependent regulation of adhesion may vary depending on cell type.

ENCCs displayed fewer FAs on VN than on FN. They recruited much more β1-integrins than β3-integrins to their adhesion sites when cultured on VN. EDN3 increased the number of β1-integrin-mediated adhesions but not the β3-integrin-mediated adhesions on VN. This indicates that EDN3 probably acts by reinforcing β1-integrin-mediated adhesions and not by changing the ENCC adhesion mode on ECM-coated surfaces.

*Edn3*^*ls/ls*^ENCCs express β1-integrins, but display fewer FAs than their control counterpart *in vitro,* on FN. This suggests that mutant ENCCs may exhibit a cell autonomous adhesion defect. This adhesion defect detected *in vitro* may be worse *in vivo*. It may synergize with the effect of EDN3 depletion in altering the ENCC environment, such as by changing the ECM composition in the surrounding tissue as described in *Edn3*^*ls/ls*^mutants^15^.

Interactions between *Edn3* and *Sox10*^11^,^37^ and between *Sox10* and *Zfhx1b*^46^ control ENS progenitor survival and differentiation. We also demonstrated the interplay between *Sox10* and β1-integrins during ENS development^38^, suggesting that integrins may act to modify the ENS phenotype as shown for the gene encoding the adhesion molecule L1CAM^47^,^48^. Here, we highlight a new genetic interaction between *Edn3* and *Itgb1* during ENS development, both in regulating gut colonization and organising the ganglia network. β1^null^;Edn3^null^ ENCCs display altered ECM adhesion, increased aggregation, and reduced migration speed and persistence of movement compared to the other mutants. These alterations were already described for β1^null^ ENCCs but were more pronounced in β1^null^;Edn3^null^ guts. Some β1^null^;Edn3^null^ ENCCs were static or formed clusters blocked at the stomach exit or half-proximal part of the midgut. The double heterozygous β1^het^;Edn3^het^ did not present ENS phenotype but in the context of *Itgb1* or *Edn3* heterozygosity the null mutation for the other gene produced aganglionosis indicating that the strength of the genetic interaction between these two genes is relatively weak. Of note, comparison of the enteric phenotype of non-conditional and conditional mutants revealed that three of the seven analysed β1^neo^;Edn3^null^ embryos presented severe defects relative to the single mutants, whereas we did not observe this for β1^het^;Edn3^null^ embryos. The difference between the two genotypes is that the former is heterozygous for *Itgb1* deletion in both ENCCs and the surrounding cells whereas the latter is heterozygous for *Itgb1* deletion only in ENCCs. This argues for both β1-integrin-mediated ENCC autonomous and non-autonomous effects in regulating ENS development in the *Edn3*^*ls/ls*^ context.

We also evidenced defects at E11.5 during the ENS development for β1^null^;Edn3^null^ double mutants with a migratory front located in the proximal stomach while in single mutants the migratory front is located in the distal midgut. Thus, together with the altered migratory properties observed at E12.5, results obtained at E11.5 highlight that genetic interaction between the two loci may rely on cooperative activity or on the successive requirement of these two molecules during various steps of ENS development. Previous findings showed that early depletion of vagal NCCs during their migration from the neural tube to the foregut^49^,^50^ or early vagal NCCs apoptosis^37^ impacts on ENS development and produces similar defects in the progression of ENCC migratory front. It would be interesting to check whether an increased apoptosis of vagal NCCs en route to the foregut occurs in the double mutant. Such effect could impact on the enteric progenitor pool contributing to the defective gut colonization.

In the developing gut, ENCCs are subjected to EDN3 signalling which inhibits PKA activity and favours RAC activation as well as lamellipodia formation at the cell front^13^. We show that under normal conditions, where ENCCs express β1-integrins, EDN3 plays a role in increasing their adhesion properties. This is consistent with the observation of ENCC rounding after treatment of embryonic guts with BQ788^29^,^30^. This process probably occurs all along the gut and especially in the caecum, which is enriched in EDN3 and ECM components including FN, TNC, and VN. The combined action of EDN3 on RAC activation, lamellipodia dynamics, and β1-integrin-mediated adhesion help to support the interaction of ENCCs with the ECM, cell spreading, and polarization to ensure proper gut colonization.

The elasticity of the environment plays a crucial role in modifying cell adhesive and migratory properties, as well as progenitor cell differentiation programs^51^,^52^,^53^. β1-integrins are mechanoreceptors that control cell sensing to external tissue elasticity^54^ and play an important role in controlling cell stem fate. ENCCs modulate their migration in response to the mechanical properties of their environment^55^. The activity of EDN3 on β1-integrin-mediated adhesion may regulate the ability of ENCCs to sense the mechanical properties of the gut tissue during ENS development.

Altogether, our results highlight the genetic interaction between *Itgb1* and *Edn3* that controls proper ENS development and the role of EDN3 in controlling ENCC adhesion. They underscore the importance of the autonomous adhesion properties of ENCCs in response to EDN3, which operate in concert with a β1-integrin-dependent non-autonomous mechanism to modulate the properties of the ENCC environment. Our results may help to improve our understanding of the mechanisms that couple biochemical and mechanical cues of the environment in the control of the survival, proliferation, and fate determination of enteric progenitors.

## Material and Methods

### Animal models, mouse maintenance and genotyping

Mouse models used in this study are as follows: *lethal spotted Edn3*^*ls*^^19^,^37^, Ht-PA::Cre^56^, *beta1*^*neo/*+^ carrying one null *Itgb1* allele^57^, Ht-PA::Cre;*beta1*^*neo/*+ *32*^and *beta1*^*fl/fl*^;R26RYFP mice carrying *Itgb1* floxed and YFP reporter alleles^5^,^31^,^58^, referred to hereafter as *beta1*^*fl/fl*^. Mouse models were used to produce embryos with genotypes of interest. The *beta1*^*neo/*+^ and *beta1*^*fl/fl*^ mice were crossed with *Edn3*^*ls/*+^ mice to produce *beta1*^*neo/*+^;*Edn3*^*ls/*+^ and *beta1*^*fl/fl*^;*Edn3*^*ls/*+^ mice. These mice were crossed to produce six additional progeny genotypes: *beta1*^+*/fl*^*;Edn3*^+/+^ control*, beta1*^*neo/fl*^*;Edn3*^+/+^ heterozygous for *Itgb1, beta1*^+*/fl*^;*Edn3*^*ls/*+^ heterozygous for the *Edn3, beta1*^*neo/fl*^;*Edn3*^*ls/*+^ double heterozygous, *beta1*^+*/fl*^;*Edn3*^*ls/ls*^ *Edn3* mutant, and the *beta1*^*neo/fl*^;*Edn3*^*ls/*ls^ mutant. In addition, Ht-PA::Cre;*beta1*^*neo/*+^ and *beta1*^*fl/fl*^ were crossed with *Edn3*^*ls/*+^ mice to produce Ht-PA::Cre;*beta1*^*neo/*+^*;Edn3*^*ls/*+^ and *beta1*^*fl/fl*^;*Edn3*^*ls/*+^ animals. These mice were then crossed to generate the six following progeny genotypes: Ht-PA::Cre;*beta1*^*fl/*+^ heterozygous for *Itgb1* in NCC^24^, Ht-PA::Cre;*beta1*^*fl/neo*^mutant for *Itgb1* in NCC and heterozygous in the other tissues, Ht-PA::Cre;*beta1*^*fl/*+^*;Edn3*^*ls/*+^ and Ht-PA::Cre;*beta1*^*fl/neo*^*;Edn3*^*ls/*+^ that are also heterozygous for the *Edn3*^*ls*^ mutation, and Ht-PA::Cre;*beta1*^*fl/*+^*;Edn3*^*ls/ls*^ and Ht-PA::Cre;*beta1*^*fl/neo*^*;Edn3*^*ls/ls*^ that are also homozygous for the *Edn3*^*ls*^ mutation. All exhibit YFP and β-galactosidase expression in NCCs. The crosses, genotypes generated, and corresponding classes of each genotype are presented in Table 1.

Fertilized chicken eggs (Gallus Gallus) from a commercial source (Morizeau, Dangers, France) were incubated at 38 °C in a humidified incubator. Embryos were staged according to somite numbers and to the Hamburger and Hamilton staging chart.

Experiments were performed in accordance with the ethical guidelines of INSERM and the CNRS. The protocol was approved by the Committee on the Ethics of Animal Experiments of the Institut Curie (National registration number: #118), and The Comité National de Réflexion Ethique sur l′Expérimentation Animale (C2EA -16).

### Organotypic cultures and immunostaining

2D-cultures of mouse midgut explants on ECM-coated surfaces were performed as described previously^5^. Except when specified, the midguts were collected from Ht-PA::Cre; *beta1*^*fl/*+^ embryos (control) at stages E11.5 or E12.5 and cultured in a culture medium composed of DMEM-F12 (Invitrogen) supplemented with glutamine (Gibco 25030–081, 1/100), penicillin-streptomycin (Gibco 15140–122, 1/100) and ITS (insulin, transferrin, selenium solution, Invitrogen; 1/100). This makes it possible to easily distinguish ENCCs (YFP^+^) from mesenchymal cells. For treated cultures, EDN3 (VWR, France) was used at 100 nM or 1 μM final concentration and compared to the control condition, hereafter referred as [-EDN3] in the figures. For the kinetics analyses, the medium of overnight cultures was changed with medium containing EDN3 [+EDN3] or solvent [−EDN3] at the same dilution as the EDN3 solution. We detected similar ENCC behaviour in the culture medium containing solvent or not. For chick 2D-cultures, midgut rings were collected at stage E6.5 and cultured as described above.

Cultures were performed on a surface coated either with fibronectin (FN, F1141, Sigma) at 20 μg/ml, vitronectin (VN, 2348-vn-100, R&D systems) at 10 μg/ml, or FN + Tenascin-C (TNC) (CC118, Merck Millipore), each at 10 μg/ml. Cultures were fixed and immunostained with specific antibodies directed against Sox10 (enteric progenitor marker), YFP, activated β1-integrin (β1*), and paxillin (a focal adhesion (FA) marker) or labelled with phalloidin. For WAVE2 immunostaining, cultures were fixed with 10% Trichloroacetic acide and DMEM solution for 20 minutes at 4 °C, rinsed with PBS and incubated for 3 min in PBS, BSA (3 mg/ml) and 0,1% triton. To analyse phalloidin and WAVE2 stainings, two independent experiments were performed for each staining.

*Ex-vivo* cultures of guts were carried out as described previously^5^. Whole guts were cultured in culture medium supplemented with 5% horse serum (Babco). Cell cultures and TUJ1 whole-mount immunostainings were performed as described previously^8^,^37^. Preparation of samples and immunostaining on paraffin and cryosections of embryos and embryonic guts were performed as described previously^32^,^59^.

The antibodies used are listed in Table S2.

### Video time-lapse imaging and image acquisitions

2D-cultures of midgut explants were incubated overnight on FN. They were then imaged for 10 min before, and immediately after, the medium change with one containing EDN3 or solvent (control medium) for 30 min. Acquisitions were performed with one image taken every 20 s mouse ENCCs can be distinguished from mesenchymal cells by their YFP expression. Chick ENCCs can be distinguished from mesenchymal cells due to a brief treatment in culture with an NC1 antibody directly coupled to a fluoprobe using LIGHTNING-LINK FLUOPROBES 647 H (Interchim). Two independent experiments were performed for both mouse and chick cultures.

Time-lapse imaging of *ex-vivo* cultures of guts were carried out as described previously^5^ on whole guts cultured in culture medium supplemented with 5% horse serum (Babco).

Image acquisitions were performed at the Nikon Imaging Centre of the Institut Curie-CNRS (NIMCE@IC-CNRS) and the IMRB microscope facility. Analysis of ENCC cultures were performed using both inverted confocal A1-R Nikon and Zeiss Axio Observer Z1 microscopes. WAVE2 immunostaining images were recorded using both epifluorescence Nikon Eclipse 90i Upright and Zeiss AxioImager M2 microscopes. Image acquisitions of whole mount TUJ1 stainings and video-time lapses of 2D cultures, *ex-vivo* gut cultures, and YFP image acquisitions were carried out as previously described^28^ using a Nikon Eclipse Ti confocal and inverted microscope.

### Quantification and statistical analysis

Quantifications of FAs were performed as follows: for each experiment, nine images of labelled ENCC clusters were taken randomly at the periphery over three different gut explants per condition. Several ENCCs were visible on each image as well as several mesenchymal cells. Signals corresponding to mesenchymal cells FAs were removed manually. ENCCs FAs were then segmented by manual thresholding using ImageJ. For each image, the area and Feret’s diameter (indicating the FA length along its most elongated direction) of each single FA were measured using ImageJ Analyse Particle plugin as described previously^38^. The average number of FAs displayed by ENCC corresponds to the mean of all the ratios, obtained for each image, between the total number of FAs quantified in the image and the number of ENCCs present on the same image. To analyse the effect of EDN3 on ENCCs adhesion, three independent experiments were performed for each condition (FN, FN + TNC and VN) and two independent experiments were performed to analyse the kinetics response of ENCCs to EDN3. For *Edn3*^*ls/ls*^ and *Edn3*^+/+^ embryos, five guts of each were analysed in total over three independent experiments. Graphs associated with each figures and Table S1 present the pool of the independent experiments performed. Mann-Whitney-Wilcoxon statistical tests were performed using R. The total number of ENCCs imaged and analysed over the independent experiments is indicated in the figure legend.

To quantify ENCC density, the area of gut tissue devoid of YFP^+^ cells (ENCCs) and TUJ1^+^ cells (neurons) was analysed on two guts of each genotype collected from different offsprings and the proportion of ENCC-free area per image determined as described previously^28^. Segmentation was applied to the maximum intensity projection of 3–6 confocal slices taken from stained guts using a self-developed ImageJ macro based on K-means clustering^60^. Statistical analyses of the ENS network and ENCC-free areas in guts were performed using the Kruskal Wallis test for multiple comparisons with Matlab.

Principal component analysis (PCA) was carried out as described previously^28^ based on texture analysis of the segmentation. PCA allows either the detection of distinct network organisations for which their position in the PCA space is separated by a decision line or of similar populations for which their dots are in the same zone of PCA space.

For *ex-vivo* gut cultures analyses, individual ENCCs were manually tracked using Metamorph 7.7.3.0 software to determine their speed of locomotion and persistence (the straightness of the trajectory, obtained by dividing the distance of the straight line between the initial and final point of the tracked cell by the total distance covered by the cell), as described previously^5^. Two independent experiments with embryos of different offsprings were analysed. Pairwise comparisons using Student’s *t* test with Holm correction of the p-value was performed using R.

P-values: not significant (n.s.), * < 0.05; ** < 0.01; *** < 0.001.

## Additional Information

**How to cite this article**: Gazquez, E. *et al*. Endothelin-3 stimulates cell adhesion and cooperates with β1-integrins during enteric nervous system ontogenesis. *Sci. Rep.* **6**, 37877; doi: 10.1038/srep37877 (2016).

**Publisher's note:** Springer Nature remains neutral with regard to jurisdictional claims in published maps and institutional affiliations.

## Supplementary Material

Supplementary Movie 1

Supplementary Movie 2

Supplementary Figure S1

Supplementary Figure S2

Supplementary Figure S3

Supplementary Figure S4

Supplementary Figure S5

Supplementary Figure S6

Supplementary Figure S7

Supplementary Figure S8

Supplementary Tables and Legends

## Figures and Tables

**Table 1:** Crossing strategies and genotypes generated for non-conditional and conditional mutants.

Crossing strategy	Genotypes	Referred to in the text as:
For non conditional mutants:	*beta1*^+*/fl*^*;Edn3*^+/+^	control
	*beta1*^*neo/fl*^*;Edn3*^+/+^	β1^neo^
*beta1*^*neo/*+^*;Edn3*^*ls/*+^	*beta1*^+*/fl*^;*Edn3*^*ls/*+^	Edn3^het^
*X beta1*^*fl/fl*^;*Edn3*^*ls/*+^	*beta1*^*neo/fl*^;*Edn3*^*ls/*+^	β1^neo^;Edn3^het^
	*beta1*^+*/fl*^;*Edn3*^*ls/ls*^	Edn3^null^
	*beta1*^*neo/fl*^;*Edn3*^*ls/ls*^	β1^neo^;Edn3^null^
For conditional mutants:	Ht-PA::Cre;*beta1*^*fl/*+^	β1^het^
	Ht-PA::Cre;*beta1*^*fl/*+^*;Edn3*^*ls/*+^	β1^het^;Edn3^het^
Ht-PA::Cre;*beta1*^*neo/*+^*;Edn3*^*ls/*+^	Ht-PA::Cre;*beta1*^*fl/neo*^	β1^null^
X*beta1*^*fl/fl*^;*Edn3*^*ls/*+^	Ht-PA::Cre;*beta1*^*fl/*+^*;Edn3*^*ls/ls*^	β1^het^;Edn3^null^
	Ht-PA::Cre;*beta1*^*fl/neo*^*;Edn3*^*ls/*+^	β1^null^;Edn3^het^
	Ht-PA::Cre;*beta1*^*fl/neo*^*;Edn3*^*ls/ls*^	β1^null^;Edn3^null^
	*Edn3*^*ls/ls*^	*Edn3*^*ls/ls*^
	*beta1*^*fl/fl*^;R26RYFP	*beta1*^*fl/fl*^

## References

[b1] Lake J. I. & Heuckeroth R. O. Enteric nervous system development: migration, differentiation, and disease. Am J Physiol Gastrointest Liver Physiol 305, G1–24, doi: 10.1152/ajpgi.00452.2012 (2013). 23639815 PMC3725693

[b2] Ames R. S. . Identification of a selective nonpeptide antagonist of the anaphylatoxin C3a receptor that demonstrates antiinflammatory activity in animal models. J Immunol 166, 6341–6348 (2001). 11342658 10.4049/jimmunol.166.10.6341

[b3] Simpson M. J., Zhang D. C., Mariani M., Landman K. A. & Newgreen D. F. Cell proliferation drives neural crest cell invasion of the intestine. Developmental Biology 302, 553–568, doi: 10.1016/j.ydbio.2006.10.017 (2007). 17178116

[b4] Hackett-Jones E. J., Landman K. A., Newgreen D. F. & Zhang D. On the role of differential adhesion in gangliogenesis in the enteric nervous system. Journal of theoretical biology 287, 148–159, doi: 10.1016/j.jtbi.2011.07.013 (2011). 21816161

[b5] Breau M. A., Dahmani A., Broders-Bondon F., Thiery J. P. & Dufour S. Beta1 integrins are required for the invasion of the caecum and proximal hindgut by enteric neural crest cells. Development 136, 2791–2801 (2009). 19633172 10.1242/dev.031419

[b6] Rauch U. & Schafer K. H. The extracellular matrix and its role in cell migration and development of the enteric nervous system. Eur J Pediatr Surg 13, 158–162 (2003). 12939699 10.1055/s-2003-41265

[b7] Young H. M. . GDNF Is a Chemoattractant for Enteric Neural Cells. Developmental Biology 229, 503–516, doi: 10.1006/dbio.2000.0100 (2001). 11150245

[b8] Barlow A., de Graaff E. & Pachnis V. Enteric nervous system progenitors are coordinately controlled by the G protein-coupled receptor EDNRB and the receptor tyrosine kinase RET. Neuron 40, 905–916 (2003). 14659090 10.1016/s0896-6273(03)00730-x

[b9] Nagy N. & Goldstein A. M. Endothelin-3 regulates neural crest cell proliferation and differentiation in the hindgut enteric nervous system. Developmental Biology 293, 203–217, doi: 10.1016/j.ydbio.2006.01.032 (2006). 16519884

[b10] Leibl M. A. . Expression of endothelin 3 by mesenchymal cells of embryonic mouse caecum. Gut 44, 246–252 (1999). 9895385 10.1136/gut.44.2.246 PMC1727386

[b11] Bondurand N., Natarajan D., Barlow A., Thapar N. & Pachnis V. Maintenance of mammalian enteric nervous system progenitors by SOX10 and endothelin 3 signalling. Development 133, 2075–2086, doi: 10.1242/dev.02375 (2006). 16624853

[b12] Wu J. J., Chen J. X., Rothman T. P. & Gershon M. D. Inhibition of *in vitro* enteric neuronal development by endothelin-3: mediation by endothelin B receptors. Development 126, 1161–1173 (1999). 10021336 10.1242/dev.126.6.1161

[b13] Goto A. . GDNF and endothelin 3 regulate migration of enteric neural crest-derived cells via protein kinase A and Rac1. J Neurosci 33, 4901–4912, doi: 10.1523/JNEUROSCI.4828-12.2013 (2013). 23486961 PMC6618995

[b14] Lee H. The endothelin receptor-B is required for the migration of neural crest-derived melanocyte and enteric neuron precursors. Developmental Biology 259, 162–175, doi: 10.1016/s0012-1606(03)00160-x (2003). 12812796

[b15] Payette R. F. . Accumulation of components of basal laminae: association with the failure of neural crest cells to colonize the presumptive aganglionic bowel of ls/ls mutant mice. Dev Biol 125, 341–360 (1988). 3338619 10.1016/0012-1606(88)90217-5

[b16] Rothman T. P. . Increased expression of laminin-1 and collagen (IV) subunits in the aganglionic bowel of ls/ls, but not c-ret *−/−* mice. Dev Biol 178, 498–513 (1996). 8812145 10.1006/dbio.1996.0234

[b17] Rothman T. P., Goldowitz D. & Gershon M. D. Inhibition of migration of neural crest-derived cells by the abnormal mesenchyme of the presumptive aganglionic bowel of ls/ls mice: analysis with aggregation and interspecies chimeras. Developmental biology 159, 559–573, doi: 10.1006/dbio.1993.1264 (1993). 8405679

[b18] Hosoda K. . Targeted and natural (piebald-lethal) mutations of endothelin-B receptor gene produce megacolon associated with spotted coat color in mice. Cell 79, 1267–1276 (1994). 8001159 10.1016/0092-8674(94)90017-5

[b19] Baynash A. . Interaction of endothelin-3 with endothelin-B receptor is essential for development of epidermal melanocytes and enteric neurons. Cell 79, 1277–1285 (1994). 8001160 10.1016/0092-8674(94)90018-3

[b20] Druckenbrod N. R., Powers P. A., Bartley C. R., Walker J. W. & Epstein M. L. Targeting of endothelin receptor-B to the neural crest. genesis 46, 396–400, doi: 10.1002/dvg.20415 (2008). 18693272 PMC2610478

[b21] Kapur R. P., Yost C. & Palmiter R. D. A transgenic model for studying development of the enteric nervous system in normal and aganglionic mice. Development 116, 167–175 (1992). 1483385 10.1242/dev.116.Supplement.167

[b22] Kruger G. M. . Neural crest stem cells persist in the adult gut but undergo changes in self-renewal, neuronal subtype potential, and factor responsiveness. Neuron 35, 657–669 (2002). 12194866 10.1016/s0896-6273(02)00827-9 PMC2728576

[b23] Iwashita T., Kruger G. M., Pardal R., Kiel M. J. & Morrison S. J. Hirschsprung disease is linked to defects in neural crest stem cell function. Science 301, 972–976 (2003). 12920301 10.1126/science.1085649 PMC2614078

[b24] Breau M. A. . Lack of beta1 integrins in enteric neural crest cells leads to a Hirschsprung-like phenotype. Development 133, 1725–1734, doi: 10.1242/dev.02346 (2006). 16571628

[b25] Brizzi M. F., Tarone G. & Defilippi P. Extracellular matrix, integrins, and growth factors as tailors of the stem cell niche. Current opinion in cell biology 24, 645–651, doi: 10.1016/j.ceb.2012.07.001 (2012). 22898530

[b26] Hynes R. O. Integrins: bidirectional, allosteric signaling machines. Cell 110, 673–687 (2002). 12297042 10.1016/s0092-8674(02)00971-6

[b27] Larsen M., Artym V. V., Green J. A. & Yamada K. M. The matrix reorganized: extracellular matrix remodeling and integrin signaling. Current Opinion in Cell Biology 18, 463–471, doi: 10.1016/j.ceb.2006.08.009 (2006). 16919434

[b28] Broders-Bondon F., Paul-Gilloteaux P., Carlier C., Radice G. L. & Dufour S. N-cadherin and beta1-integrins cooperate during the development of the enteric nervous system. Dev Biol 364, 178–191, doi: 10.1016/j.ydbio.2012.02.001 (2012). 22342243

[b29] Young H. M. . Colonizing while migrating: How do individual enteric neural crest cells behave? BMC Biology 12, 23, doi: 10.1186/1741-7007-12-23 (2014). 24670214 PMC4101823

[b30] Druckenbrod N. R. & Epstein M. L. Age-dependent changes in the gut environment restrict the invasion of the hindgut by enteric neural progenitors. Development 136, 3195–3203, doi: 10.1242/dev.031302 (2009). 19700623

[b31] Srinivas S. . Cre reporter strains produced by targeted insertion of EYFP and ECFP into the ROSA26 locus. BMC Dev Biol 1, 4 (2001). 11299042 10.1186/1471-213X-1-4 PMC31338

[b32] Pietri T. . Conditional beta1-integrin gene deletion in neural crest cells causes severe developmental alterations of the peripheral nervous system. Development 131, 3871–3883, doi: 10.1242/dev.01264 (2004). 15253938

[b33] Krause M. & Gautreau A. Steering cell migration: lamellipodium dynamics and the regulation of directional persistence. Nature reviews. Molecular cell biology 15, 577–590, doi: 10.1038/nrm3861 (2014). 25145849

[b34] Hall A. Rho GTPases and actin cytoskeleton. Science 279, 509–514 (1998). 9438836 10.1126/science.279.5350.509

[b35] Vicente-Manzanares M., Choi C. K. & Horwitz A. R. Integrins in cell migration - the actin connection. Journal of Cell Science 122, 1473–1473, doi: 10.1242/jcs.052894 (2009). PMC2714416 19118212

[b36] Stephens L. E. . Deletion of beta 1 integrins in mice results in inner cell mass failure and peri-implantation lethality. Genes Dev 9, 1883–1895 (1995). 7544312 10.1101/gad.9.15.1883

[b37] Stanchina L. . Interactions between Sox10, Edn3 and Ednrb during enteric nervous system and melanocyte development. Developmental Biology 295, 232–249, doi: 10.1016/j.ydbio.2006.03.031 (2006). 16650841

[b38] Watanabe Y. . Sox10 and Itgb1 interaction in enteric neural crest cell migration. Developmental biology 379, 92–106, doi: 10.1016/j.ydbio.2013.04.013 (2013). 23608456

[b39] Koyama Y., Yoshioka Y., Hashimoto H., Matsuda T. & Baba A. Endothelins increase tyrosine phosphorylation of astrocytic focal adhesion kinase and paxillin accompanied by their association with cytoskeletal components. Neuroscience 101, 219–227 (2000). 11068150 10.1016/s0306-4522(00)00330-4

[b40] Lange K. . Endothelin Receptor Type B Counteracts Tenascin-C-Induced Endothelin Receptor Type A-Dependent Focal Adhesion and Actin Stress Fiber Disorganization. Cancer Research 67, 6163–6173, doi: 10.1158/0008-5472.can-06-3348 (2007). 17616673

[b41] Bagnato A. . Endothelin B receptor blockade inhibits dynamics of cell interactions and communications in melanoma cell progression. Cancer Res 64, 1436–1443 (2004). 14973117 10.1158/0008-5472.can-03-2344

[b42] Horton E. R., Astudillo P., Humphries M. J. & Humphries J. D. Mechanosensitivity of integrin adhesion complexes: role of the consensus adhesome. Experimental cell research 343, 7–13, doi: 10.1016/j.yexcr.2015.10.025 (2016). 26515553

[b43] Etienne-Manneville S. & Hall A. Rho GTPases in cell biology. Nature 420, 629–635, doi: 10.1038/nature01148 (2002). 12478284

[b44] Parsons J. T., Horwitz A. R. & Schwartz M. A. Cell adhesion: integrating cytoskeletal dynamics and cellular tension. Nat Rev Mol Cell Biol 11, 633–643, doi: 10.1038/nrm2957 (2010). 20729930 PMC2992881

[b45] Liu Y. . Autocrine endothelin-3/endothelin receptor B signaling maintains cellular and molecular properties of glioblastoma stem cells. Molecular cancer research: MCR 9, 1668–1685, doi: 10.1158/1541-7786.MCR-10-0563 (2011). 22013079 PMC3245317

[b46] Stanchina L., Van de Putte T., Goossens M., Huylebroeck D. & Bondurand N. Genetic interaction between Sox10 and Zfhx1b during enteric nervous system development. Developmental Biology 341, 416–428, doi: 10.1016/j.ydbio.2010.02.036 (2010). 20206619

[b47] Wallace A. S., Tan M. X., Schachner M. & Anderson R. B. L1cam acts as a modifier gene for members of the endothelin signalling pathway during enteric nervous system development. Neurogastroenterol Motil 23, e510–522, doi: 10.1111/j.1365-2982.2011.01692.x (2011). 21395909

[b48] Wallace A. S., Schmidt C., Schachner M., Wegner M. & Anderson R. B. L1cam acts as a modifier gene during enteric nervous system development. Neurobiology of Disease 40, 622–633, doi: 10.1016/j.nbd.2010.08.006 (2010). 20696247

[b49] Burns A. J. & Le Douarin N. M. Enteric nervous system development: analysis of selective developmental potentialities of vagal and sacral neural crest cells using quail-chick chimeras. Anat. Rec 262, 16–28 (2001). 11146425 10.1002/1097-0185(20010101)262:1<16::AID-AR1007>3.0.CO;2-O

[b50] Faure S., McKey J., Sagnol S. & de Santa Barbara P. Enteric neural crest cells regulate vertebrate stomach patterning and differentiation. Development 142, 331–342, doi: 10.1242/dev.118422 (2015). 25519241

[b51] Engler A. J., Sen S., Sweeney H. L. & Discher D. E. Matrix elasticity directs stem cell lineage specification. Cell 126, 677–689 (2006). 16923388 10.1016/j.cell.2006.06.044

[b52] Fu J. . Mechanical regulation of cell function with geometrically modulated elastomeric substrates. Nat Methods 7, 733–736 (2010). 20676108 10.1038/nmeth.1487 PMC3069358

[b53] Tenney R. M. & Discher D. E. Stem cells, microenvironment mechanics, and growth factor activation. Curr Opin Cell Biol 21, 630–635 (2009). 19615877 10.1016/j.ceb.2009.06.003 PMC2775547

[b54] Moore S. W., Roca-Cusachs P. & Sheetz M. P. Stretchy Proteins on Stretchy Substrates: The Important Elements of Integrin-Mediated Rigidity Sensing. Developmental Cell 19, 194–206, doi: 10.1016/j.devcel.2010.07.018 (2010). 20708583 PMC5319208

[b55] Chevalier N. R. . How Tissue Mechanical Properties Affect Enteric Neural Crest Cell Migration. Sci Rep. 6, 20927, 10.1038/srep20927 (2016). 26887292 PMC4757826

[b56] Pietri T., Eder O., Blanche M., Thiery J. P. & Dufour S. The human tissue plasminogen activator-Cre mouse: a new tool for targeting specifically neural crest cells and their derivatives *in vivo*. Dev Biol 259, 176–187 (2003). 12812797 10.1016/s0012-1606(03)00175-1

[b57] Fässler R. & Meyer M. Consequence of lack of b1 integrin gene expression in mice. Genes Dev. 9, 1896–1908 (1995). 7544313 10.1101/gad.9.15.1896

[b58] Potocnik A. J., Brakebusch C. & Fassler R. Fetal and adult hematopoietic stem cells require beta1 integrin function for colonizing fetal liver, spleen, and bone marrow. Immunity 12, 653–663 (2000). 10894165 10.1016/s1074-7613(00)80216-2

[b59] Delannet M. . Specific roles of the avb1, avb3, and avb5 integrins in avian neural crest cell adhesion and migration on vitronectin. Development 120, 2687–2702 (1994). 7525179 10.1242/dev.120.9.2687 PMC2710119

[b60] Dima A. A. . Comparison of segmentation algorithms for fluorescence microscopy images of cells. Cytometry A 79, 545–559, doi: 10.1002/cyto.a.21079 (2011). 21674772

