# Supplementary material for: Endothelin-3 stimulates cell adhesion and cooperates with β1-integrins during enteric nervous system ontogenesis
Source: Sci Rep. 2016 Dec 1;6:37877. doi: 10.1038/srep37877 (PMC5131347; doi:10.1038/srep37877)
Supplement: Supplementary Figure S5 [file srep37877-s7.pdf]

Endothelin-3 stimulates cell adhesion and cooperates with β1-integrins during enteric nervous system ontogenesis.

Elodie Gazquez[1,2,3], Yuli Watanabe[4,2,3], Florence Broders-Bondon[1,a], Perrine Paul-Gilloteaux[5,b], Julie Heysch[1], Viviane Baral[4,2,3], Nadège Bondurand[4,2,3] and Sylvie Dufour[1,2,3,#].

[1]Institut Curie, CNRS, UMR144, Paris, 75005, France. [2]INSERM, U955, Team 6, Créteil, 94000, France. [3]Université Paris Est, Faculté de Médecine, Créteil, 94000, France. [4]INSERM, U955, Team 11, Créteil, 94000, France. [5]Cell and Tissue Imaging Facility, PICT-IBiSA, Paris, 75005, France.

SUPPLEMENTARY FIGURE 5

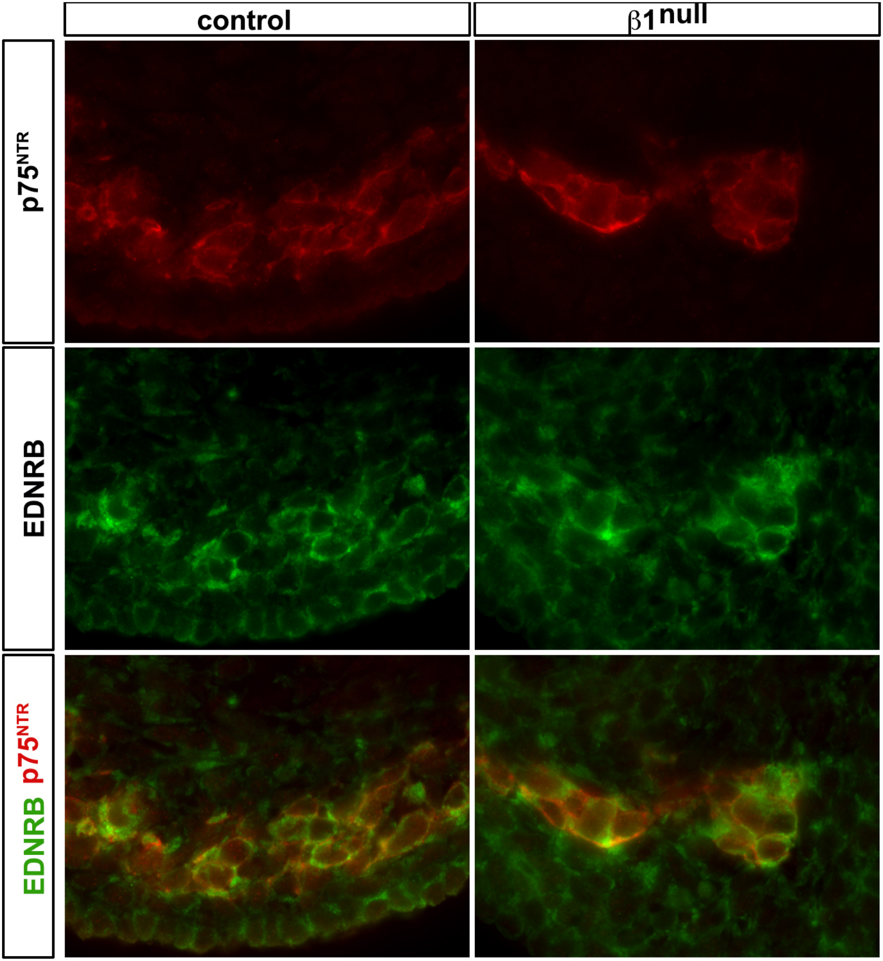
control
β1 null
p75NTR
EDNRB
EDNRB p75NTR
