# Supplementary material for: Endothelin-3 stimulates cell adhesion and cooperates with β1-integrins during enteric nervous system ontogenesis
Source: Sci Rep. 2016 Dec 1;6:37877. doi: 10.1038/srep37877 (PMC5131347; doi:10.1038/srep37877)
Supplement: Supplementary Tables and Legends [file srep37877-s11.pdf]

Endothelin-3 stimulates cell adhesion and cooperates with β1-integrins during enteric nervous system ontogenesis.

Elodie Gazquez[1,2,3], Yuli Watanabe[4,2,3], Florence Broders-Bondon[1,a], Perrine Paul-Gilloteaux[5,b], Julie Heysch[1], Viviane Baral[4,2,3], Nadège Bondurand[4,2,3] and Sylvie Dufour[1,2,3,#].

[1]Institut Curie, CNRS, UMR144, Paris, 75005, France. [2]INSERM, U955, Team 6, Créteil, 94000, France. [3]Université Paris Est, Faculté de Médecine, Créteil, 94000, France. [4]INSERM, U955, Team 11, Créteil, 94000, France. [5]Cell and Tissue Imaging Facility, PICT-IBiSA, Paris, 75005, France.

SUPPLEMENTARY MATERIALS (tables, figures legends and movies)

Supplementary Tables:

Table S1: Parameters of ENCC focal adhesions of cells cultured on different ECM proteins in the presence (+) or absence of EDN3 (-). Statistical significance relative to control condition (-EDN3); ns: not significant; p values * < 0.05; ** < 0.01; *** < 0.001.

<table>
<tr><th colspan="9">Treatment Length (-/+ EDN3)</th></tr>
<tr><th colspan="9">Fibronectin</th></tr>
<tr><th rowspan="2">FA β1*+</th><th colspan="2">24h</th><th colspan="2">30min</th><th colspan="2">1h</th><th colspan="2">3h</th></tr>
<tr><th>-</th><th>+</th><th>-</th><th>+</th><th>-</th><th>+</th><th>-</th><th>+</th></tr>
<tr><td>Area (µm2)</td><td>0.239 ±0.002</td><td>0.330 ±0.003***</td><td>0.17 ±0.003</td><td>0.21 ±0.004***</td><td>0.16 ±0.001</td><td>0.18 ±0.002 ns</td><td>0.15 ±0.003</td><td>0.21 ±0.002***</td></tr>
<tr><td>Feret ∅ (µm)</td><td>0.784 ±0.003</td><td>0.929 ±0.004***</td><td>0.68 ±0.006</td><td>0.77 ±0.006**</td><td>0.65 ±0.003</td><td>0.73 ±0.003***</td><td>0.63 ±0.004</td><td>0.76 ±0.003**</td></tr>
<tr><th rowspan="2">FA Pax+</th><th colspan="2">24h</th><th colspan="2">30min</th><th colspan="2">1h</th><th colspan="2">3h</th></tr>
<tr><th>-</th><th>+</th><th>-</th><th>+</th><th>-</th><th>+</th><th>-</th><th>+</th></tr>
<tr><td>Area (µm2)</td><td>0.231 ±0.004</td><td>0.311 ±0.002***</td><td>0.19 ±0.003</td><td>0.29 ±0.006***</td><td>0.18 ±0.003</td><td>0.21 ±0.003ns</td><td>0.16 ±0.003</td><td>0.27 ±0.004***</td></tr>
<tr><td>Feret ∅ (µm)</td><td>0.797 ±0.005</td><td>0.922 ±0.002***</td><td>0.76 ±0.005</td><td>0.89 ±0.007**</td><td>0.72 ±0.005</td><td>0.79 ±0.006**</td><td>0.68 ±0.005</td><td>0.85 ±0.006**</td></tr>
<tr><th rowspan="3">FA β1*+</th><th colspan="4">Fibronectin+ Tenascin-C</th><th colspan="4">Vitronectin</th></tr>
<tr><th colspan="8">24h</th></tr>
<tr><td colspan="2">-</td><td colspan="2">+</td><td colspan="2">-</td><td colspan="2">+</td></tr>
<tr><td>Area (µm2)</td><td colspan="2">0.17 ±0.01</td><td colspan="2">0.20 ±0.01***</td><td colspan="2">0.17 ±0.04</td><td colspan="2">0.20 ±0.03***</td></tr>
<tr><td>Feret ∅ (µm)</td><td colspan="2">0.68 ±0.02</td><td colspan="2">0.76 ±0.02**</td><td colspan="2">0.67 ±0.07</td><td colspan="2">0.72 ±0.03**</td></tr>
<tr><th rowspan="3">FA Pax+</th><th colspan="4">Fibronectin+ Tenascin-C</th><th colspan="4">Vitronectin</th></tr>
<tr><th colspan="8">24h</th></tr>
<tr><td colspan="2">-</td><td colspan="2">+</td><td colspan="2">-</td><td colspan="2">+</td></tr>
<tr><td>Area (µm2)</td><td colspan="2">0.27 ±0.003</td><td colspan="2">0.33 ±0.003***</td><td colspan="2">0.17 ±0.06</td><td colspan="2">0.24 ±0.04***</td></tr>
<tr><td>Feret ∅ (µm)</td><td colspan="2">0.861 ±0.004</td><td colspan="2">0.950 ±0.004***</td><td colspan="2">0.70 ±0.01</td><td colspan="2">0.82 ±0.001**</td></tr>
</table>

Table S2: List of the antibodies and fluorescently-coupled reagents used in the study.

Antibody or reagent	Host	Source	dilution
Sox10 (N20)	Goat	Santa Cruz	1/50e
β1 integrin (Mab1997)	Rat	Chemicon	1/100e
activated β1 integrin (9EG7)	Rat	Pharmingen	1/200e
chick β1 integrin (clES66)	Rat	kindly provided by Jean-Loup Duband	1/500e
activated chick β1 integrin (TASK)	Mouse	Chemicon	1/100e
P75NTR	Rabbit	Promega	1/250e
β3 tubulin (TUJ1)	Mouse	Covance	1/1000e
β3 integrin (01861D)	Armenian Hamster	Pharmingen	1/100e
Paxillin	Mouse	BD transduction laboratories	1/200eme
Wave2	Rabbit	Alexis Gautreau	1/500e
YFP (A11122)	Rabbit	Molecular Probes	1/100e
EDNRB (8Z11)	Mouse	IBL	1/50e
NC1	Mouse	In-House	1/1000e
Secondary antibody (A488, A555, A647)	Donkey	Molecular Probes	1/1000e
Secondary antibody (A488, Cy3, Cy5)	Donkey	Jackson ImmunoResearch Laboratories	1/300e
Phalloidin A488		Molecular Probes	1/200e

Supplementary Figures:

Figure S1: Effect of EDN3 on ENCC FAs after 24h of culture on fibronectin+tenascin-C. a - Confocal images of control E12.5 gut explants cultured 24 h with or without EDN3 and immunolabeled for Sox10 and YFP (ENCCs left panels), activated β1-integrin (β1*), or paxillin. Merged images of β1*integrin and paxillin immunostaining are shown in the right panels. m: Mesenchymal cells ($Sox10^{-}/YFP^{-}$ cells). Scale bar = 10 µm. b- Quantification of the average number of FAs per ENCC. The total number of cells analysed for β1*integrin staining was n = 744 and n = 617 and for paxillin staining n = 861 and n = 722, under control and EDN3 conditions, respectively. Error bars indicate SEM; ***$p < 0.001$.

Figure S2: Effect of EDN3 treatment on activated β1 and β3 integrins FAs of ENCCs cultured *in vitro* onto vitronectin. a and c, Confocal images of control E12.5 gut explants cultured 24 h on VN and immunolabeled for Sox10 and YFP (ENCCs, white, left panels), activated β1-integrin (β1*, red, upper panels), β3 integrin (β3, red, lower panels), and paxillin (green). The letter m refers to mesenchymal cells ($Sox10^-/YFP^-$ cells). Merged images for activated β1-integrin or β3-integrin (red) and paxillin (green) are shown in the right panels. Scale bars = 10 µm. b- Quantification of the average number of FAs per ENCC. The total number of cells analysed over three independent experiments was n = 398 and n = 640, under control and EDN3 conditions, respectively. Error bars indicate the SEM; **p < 0.01; ***p < 0.001).

Figure S3: Effect of EDN3 treatment on chick ENCCs cultured *in vitro*. Confocal images of E6 chick gut explant cultures treated or not with EDN3 for 30 min, 1 h, and 3 h, and immunolabeled for NC1 (white), activated β1-integrin (β1*, red), and phalloidin (green) to visualize F-actin and Dapi (blue) to stain the nuclei. Mesenchymal cells ($NC1^-$ cells) are indicated by (m). Arrows point to the FAs in EDN3-treated cells in the β1* images. Arrows point to cortical and branched actin (revealed by phalloidin staining) in lamellipodia of untreated and EDN3-treated ENCCs, respectively.

Figure S4: Analysis of ENCC protrusion dynamics. Fluorescent images taken after overnight culture of a mouse gut explant on FN in control medium. They permit the visualization of mouse ENCC (YFP+) on movies 1 (a) and 2 (b) before time-lapse imaging. The ENCC shown on the movies is indicated by an asterisk and in the insert in phase contrast). Scale bar = 10 µm

Figure S5: EDNRB expression in $\beta1^{null}$ mutant ENCCs. Immunostaining of E12.5 control and $\beta1^{null}$ caecum cryosections for $P75^{NTR}$ (upper panels) or EDNRB (middle panels). The merged images for $P75^{NTR}$ (red) and EDNRB (green) staining are presented in the lower panels.

Figure S6: Itgb1 heterozygosity influences on ENS development in the context of *Edn3* mutation.

Whole-mount TUJ1 immunostaining performed on E14.5 guts from control, $\beta1^{neo}$, $Edn3^{het}$, $\beta1^{neo}$;$Edn3^{het}$, $Edn3^{null}$ and $\beta1^{neo}$;$Edn3^{null}$ embryos. Panels (left to right) show staining in the distal stomach, proximal, middle, and distal portions of the midgut, caecum, and colon for the indicated genotypes. A schematic representation of the gut with the lines and perpendicular arrows indicating the extent of colonization for each class of genotyped embryos is shown at the bottom. The number of embryos presenting with a defined defect are indicated to the left of each arrow.

Figure S7: PCA analysis of the ENS network of the various progeny genotypes. Scatter-plot diagrams in the PCA space of confocal data sets for E14.5 ENS organisation in the second half of the small intestine from the non-conditional and conditional mutants. A colour code associated with the various genotypes is indicated in the box. The black decision line discriminates between similar or different neuronal network organisations. The $\beta1^{neo}$; $Edn3^{null}$ mutant (black dots) displays a distinct organisation of ENS neuronal network relative to the others.

Figure S8: ENS defect of the various progeny genotypes at E11.5. Whole-mount X-Gal staining of E11.5 embryonic gut of indicated genotypes. Note the severe migration delay in the $\beta1^{null}$; $Edn3^{null}$. Number of guts analysed: $\beta1^{het}$;$Edn3^{het}$ guts (n = 6), $\beta1^{null}$ (n = 5), $\beta1^{het}$; $Edn3^{null}$ (n = 3), $\beta1^{null}$; $Edn3^{het}$ (n = 2) and $\beta1^{null}$;$Edn3^{null}$ (n =2).

<u>Supplementary Movies</u>

Movie 1: Dynamics of ENCC protrusions after EDN3 treatment. Time-lapse imaging was performed for 30 minutes immediately after changing the medium of mouse gut explant culture to one containing EDN3. It shows the dynamics of ENCC protrusions and the induction of a sustained growth of a lamellipodia after 10 min of EDN3 treatment. Phase contrast images were taken using the 60x objective (Plan ApoVC, oil, DIC, NA1.4) every 20sec. The ENCC can be recognised by its YFP expression (see Fig. S4a). The upper right panels of Figure 2b were extracted from the video.

Movie 2: Dynamics of ENCC protrusions in control medium. Time-lapse imaging was performed for 30 minutes immediately after changing the medium of another mouse gut explant culture to one containing the solvent. We did not detect the sustained growth of a lamellipodia in these conditions. The image acquisition and video parameters are the same as those described for movie 1. The lower right panels of Figure 2b were extracted from the video.
